# Infectious Risks Related to Umbilical Venous Catheter Dwell Time and Its Replacement in Newborns: A Narrative Review of Current Evidence

**DOI:** 10.3390/life13010123

**Published:** 2022-12-31

**Authors:** Lucia Corso, Martina Buttera, Francesco Candia, Francesca Sforza, Katia Rossi, Licia Lugli, Francesca Miselli, Luca Bedetti, Cecilia Baraldi, Laura Lucaccioni, Lorenzo Iughetti, Alberto Berardi

**Affiliations:** 1 School of Pediatrics Residency, University of Modena and Reggio Emilia, 41224 Modena, Italy; 2 Neonatal Intensive Care Unit, University Hospital of Modena, 41224 Modena, Italy; 3 Pediatrics Unit, University Hospital of Modena, 41224 Modena, Italy

**Keywords:** UVC, neonates, CLABSI, infection

## Abstract

The use of umbilical venous catheters (UVCs) has become the standard of care in the neonatal intensive care unit (NICU) to administer fluids, medications and parenteral nutrition. However, it is well known that UVCs can lead to some serious complications, both mechanical and infective, including CLABSI (Central Line-Associated Bloodstream Infections). Most authors recommend removing UVC within a maximum of 14 days from its placement. However, the last Infusion Therapy Standards of Practice (INS) guidelines recommends limiting the UVC dwell time to 7 to 10 days, to reduce risks of infectious and thrombotic complications. These guidelines also suggest as an infection prevention strategy to remove UVC after 4 days, followed by the insertion of a PICC if a central line is still needed. Nevertheless, the maximum UVC dwell time to reduce the risk of CLABSI is still controversial, as well as the time of its replacement with a PICC. In this study we reviewed a total of 177 articles, found by using the PubMed database with the following search strings: “UVC AND neonates”, “(neonate* OR newborn*) AND (UVC OR central catheter*) AND (infection*)”. We also analyze the INS guidelines to provide the reader an updated overview on this topic. The purpose of this review is to give updated information on CVCs infectious risks by examining the literature in this field. These data could help clinicians in deciding the best time to remove or to replace the UVC with a PICC, to reduce CLABSIs risk. Despite the lack of strong evidence, the risk of CLABSI seems to be minimized when UVC is removed/replaced within 7 days from insertion and this indication is emerging from more recent and larger studies.

## 1. Introduction

Central venous catheters (CVCs) are commonly used in neonatal intensive care units (NICUs) to administer fluids, medications and total parenteral nutrition (TPN) to critically ill newborns.

The umbilical venous catheter (UVC), together with neonatal PICC (peripherally inserted central catheter), here simply referred as “PICC” or “ECC” (epicutaneous-caval catheter), represents one of the most utilized central venous accesses in neonatal intensive care units (NICUs).

The UVC is a secure central access. It is usually placed in the first hours of the newborn’s life, when the umbilical vein is clearly visible and accessible in the umbilical cord. After approximatively 48 h, the umbilical cord becomes dry and colonized by micro-organisms, therefore the insertion of a UVC is not recommended [1,2,3,4]. The optimal position of the UVC tip is at the junction of the inferior vena cava and right atrium, just above the diaphragm. The current gold standard technique to assess the position of the tip is ultrasonography, although in previous decades a thoracoabdominal radiograph was usually performed [5].

A PICC is an access inserted in one of the major peripheral veins of the neonate. If necessary, PICC can be placed from birth onward at any time during NICU admission. The PICC can be inserted when the placement of an UVC has failed or when an UVC is removed (both electively or because of complications), and a continuous intravenous therapy or total parenteral nutrition is still required. Common sites used for PICC insertion are the upper limbs veins (basilic, cephalic, axillary or veins of the forearm) or the lower limbs veins (small saphenous vein at the level of popliteal vein or great saphenous vein). The optimal line tip position is in the superior vena cava, at the right atrium junction, and in the inferior vena cava at the entry into the right atrium [6]. After the catheter placement, an echocardiography is usually performed to confirm the correct placement of the tip.

Despite the routine use of central venous catheters, they can be associated with both mechanical and infective complications. Central line-associated bloodstream infections (CLABSI) represent one of the most common and severe complications related to the use of an UVCs [7,8,9].

CLABSI is defined as a laboratory-confirmed bloodstream infection, not secondary to an infection at another body site, where an organism is identified, and a central line is present for at least 24 h before the event or removed 24 h prior to the event [10].

As far as concerns UVC, it is often not easy to predict the clinical course of the newborn at the time of UVC insertion, so there is a significant risk of misusing this device, especially in preterm infants. Therefore, some investigators recommend removing the UVC within a maximum of 14 days from its placement as an infection prevention strategy. However, the correlation between the dwell time of UVCs and the rate of CLABSI in infants admitted to NICU is still unclear. The last recommendations for infusion treatment recommend limiting the UVC dwell time to 7 to 10 days to reduce infectious risks and thrombotic complications [11]. It is also questioned as to whether an early replacement of UVC (within 7 days of insertion) with PICC can reduce the rate of infections.

However, complications associated with CVCs are not only infectious. In particular, regarding UVCs, non-infectious complications may occur: malposition (30% of cases), peritoneal extravasation (2.8% of cases), venous thrombosis (1.9% of cases), difficulties in removal (0.4% of cases), symptomatic thrombosis (0.4% of cases), cardiac tamponade (0.1% of cases) and pleural effusion (0.1% of cases) [12].

The purpose of this review is to provide updated information on CVCs infectious risks by examining the literature in this field. These data could help clinicians in deciding the best time to remove or to replace an UVC with a PICC to reduce risks of CLABSIs.

## 2. Materials and Methods

The studies included in this review were retrieved after searching PubMed database by using the following search strings: “UVC AND neonates”, “(neonate* OR newborn*) AND (UVC OR central catheter*) AND (infection*)”. We identified a total of 177 articles. Among these studies, we excluded those unrelated to the purpose of our study, the aim of which is to analyze the correlation between central catheter dwell time and the risk of infectious complications in newborns of any gestational age.

To obtain a full overview on this topic, we also examined the most recent available guidelines on infusion therapy in infants and cited references [11].

## 3. Results

We included 9 studies examining the timing of elective CVC replacement to reduce the risk of infections in newborns. These studies were divided according to their conclusions: studies supporting or not supporting an association between the catheter dwell time and an increased risk of CLABSI. The main results of these studies are summarized in two tables, also giving information on study their design and main conclusions.

### 3.1. Studies That Do Not Demonstrate an Association between UVC Dwell Time and Risk of CLABSI (Table 1)

A randomized controlled trial (RCT) was carried out by O’Hara and coworkers between 1998 and 2004 in a tertiary referral neonatal unit in the USA [13]. This RCT evaluated the effect of routinely removing UVCs and replacement with a percutaneous central catheter within 7 to 10 days (group 1) as compared to a longer dwell time, with replacement up to 28 days (group 2). The participants were infants of birth weight less than 1251 g who had a double-lumen UVC placed upon admission to the neonatal unit. Infants who required an UVC for exchange transfusion, with gastrointestinal abnormalities and those with congenital heart disease were excluded. Two study arms were considered. The intervention group (early planned removal) was composed by 106 infants with UVC maintenance for up to 7 to 10 days. On the 10th day, if an ongoing central line was required, UVC was removed and a PICC was placed. The control group (expectant management) was composed by 104 neonates. Among them, the UVC was removed after day 10 when it was no longer required and by 28 days at the latest. The authors found 7.4 catheter-related infections per 1000 catheter days (group 1) and 11.5 catheter-related infections per 1000 catheter days in group 2, with an overall incidence of catheter sepsis of 13% and 20%, respectively (*p* = 0.17). The time to catheter-related infection, which was the primary outcome, did not differ between the two groups. However, while the overall incidence of catheter sepsis was not significantly different between groups, the study reported more than twice incidence of infections in the long-term UVC group as compared to the group in which UVCs were replaced by percutaneous central catheters.

A main limitation of this study was the small population of neonates who were enrolled; furthermore, the maintenance of UVC up to 28 days is far longer than that is usually recommended in all the subsequent studies, limiting the generalizability of these results.

A further study was a retrospective observational study evaluating the need to replace the UVC with a PICC in order to reduce infectious complications in newborns [6]. The investigators included 232 neonates with a gestational age between 24 and 42 weeks. In total, 255 CVCs were inserted but only 203 (UVC n = 140 or PICC n = 63) met the inclusion criteria and were used for analysis. Investigators excluded infants with a CVC put in site for ECMO, placed in a hospital different from that of the study, a CVC removed within the first 24 h of life or when data were incomplete. The mean dwell time was 6.9 ± 2.7 days for UVCs and 10.2 ± 5.2 days for a PICCs. The presence of CLABSI was one of the factors considered in the study as a non-elective reason for the CVC removal. The authors did not find any significant difference in infectious complications between UVCs and PICCs if dwell time of the CVC did not exceed 14 days (*p* = 0.60). UVCs maintained in place until day 14 as compared with PICC were apparently safe, most likely indicating that the elective removal of UVCs after 7 days is unnecessary. The authors used a Cox regression method to control the numerous variables in the population examined, such as the significant differences in birth weight and gestational age between the two study populations. The main limitations of this study were the relatively small sample size and the wide range of gestational age among enrolled neonates.

Shalabi and coworkers carried out a retrospectively matched cohort study by examining 540 neonates born under 30 weeks’ gestation admitted to 29 NICUs in the Canadian Neonatal Network between January 2010 and December 2013 [14]. The purpose of the study was to compare the rates of CLABSI in neonates who received a PICC as compared to those receiving an UVC (as their primary venous access) immediately after birth. The study population was divided into three groups of infants receiving a CVC in the first day of life: group 1 (n = 180), who received a PICC; group 2 (n = 180), who received an UVC; and group 3 (n = 180), who received an UVC that was then replaced with a PICC after 4 or more days. The primary outcome of the study was to assess the number of infants with CLABSI/1000 catheter days, which was compared among the three study groups by using multivariable analyses. Although the incidence of late onset sepsis was lower among infants who received an UVC only, no significant differences were found between the three study groups (9.3 vs. 7.8 vs. 8.2/1000 catheter days, respectively). However, the study design was imperfect, firstly because it was not randomized; furthermore, differences in the CVC dwell time between the three groups were substantial; as a matter of fact, infants who received an UVC only had the shortest dwell time as compared to the two remaining groups. Consequently, the risks of infection were lower.

An observational study was carried out by Konstantinidi and coworkers at a tertiary General Hospital in Greece, over an 18-month period [15]. The aim of the study was to investigate the complications of UVC and PICC in very low birth weight (VLBW, <1500 g) infants.

Investigators enrolled 71 VLBW neonates aged less than 32 weeks’ gestation and excluded infants who had an UVC in place for less than 24 h and those who had an UVC inserted in another center.

Neonates were divided in two groups: 34 who underwent a PICC insertion (when the UVC insertion during the first 3 days of life failed) and 37 who received an UVC but never had a PICC in place. The dwell time was 11.91 ± 6.93 days (for PICC) and 10.43 ± 5.38 days (for UVC). The authors found a CLABSI risk of 2.28 (per 1000 PICC days) and 2.59 (per 1000 UVC days, *p* = 0.952). They concluded that the risks of infection do not differ among neonates receiving a PICC insertion with respect to those receiving an UVC in the first three days of life. However, the small sample size likely precluded the reliability of the results.

Finally, through an observational study, Hei investigated UVC-related infections according to birth weight in a 50-bed level II/III Chinese NICU [16]. A total of 516 neonates were divided into four groups: infants with a birth weight ≤ 2000 g, with (n = 131) and without (n = 122) an UVC, and neonates with a birth weight over 2000 g, with (n = 154) or without (n = 109) an UVC. All UVCs were removed after 7 days, and tip cultures were performed. The overall incidence of UVC-related septicemia was 13.6/1000 UVC days (9.5%). No significant differences in the overall incidence of infections were found among the four study groups. The authors concluded that the presence of an UVC does not increase the incidence of infections in NICU when a proper UVC care bundle is followed and UVC dwell time remains under 7 days. Nevertheless, the authors did not exclude that an UVC left in place for a longer period (up to 14 days) may instead lead to differences between the study groups in terms of global infectious risks.

**Table 1:** Studies showing no association between UVC dwell time and risk of CLABSI.

Study Design (Ref.)	Study Population	Main Results	Conclusions
Randomized controlled trial *Single centre* [13]	**n = 210** <1250 g Group 1 (n = 106): UVC for 7–10 days followed by a PICC Group 2 (n = 104): long- lasting UVC (up to 28 days)	Group 1: 7.4 CLABSI per 1000 catheter days Group 2: 11.5 CLABSI per 1000 catheter days	Catheter-related infections did not differ between short- and long-lasting UVC
Retrospective observational study *Single centre* [6]	**n = 232 (203 CVC)** 24 to 42 weeks Group 1: UVC only (n = 140) Group 2: PICC only (n = 63)	33 neonates presented CLABSI (21 UVC and 12 PICC) Total CLABSI 20.5 per 1000 CVC days	No significant differences in CLABSI between the UVCs and the PICCs in the first 14 days (*p* = 0.60)
Retrospective matched cohort study *Single centre* [14]	**n = 540** >30 weeks Group 1: PICC only (n = 180) Group 2: UVC only (n = 180) Group 3: UVC replaced with a PICC after > 4 days (n = 180)	Group 1: **9.3** CLABSI/1000 catheter days Group 2: **7.8** CLABSI/1000 catheter days Group 3: **8.2** CLABSI/1000 catheter days *p* > 0.05	No difference in the incidence of CLABSI between UVC or PICC (as first placement)
Observational study *Single centre* [15]	**n = 71** VLBW, <32 weeks’ gestation Group 1: PICC only (n = 34) Group 2: UVC only (n = 37)	Group 1: **2.28** CLABSI/1000 catheter days Group 1: **2.59** CLABSI/1000 catheter days *p* = 0.952	No change in the risk of infection between neonates with a PICC as compared to those with an UVC in the first 3 days of life
Observational study *Single centre* [16]	**n = 516** Group 1 (n = 131): BW ≤ 2000 g, with UVC Group 2 (n = 122): BW ≤ 2000 g, without UVC Group 3 (n = 154): BW > 2000 g, with UVC Group 4 (n = 109): BW >2000 g, without UVC	Group 1: **14.8** CLABSI/1000 catheter days Group 2: **11.7** CLABSI/1000 catheter days Group 3: **13.6** CLABSI/1000 catheter days Group 4: **7.9** CLABSI/1000 catheter days Overall incidence of **UVC-related** septicemia was **13.6**/1000 UVC days	UVC do not increase the incidence of CLABSI in NICU, when a proper UVC care bundles is followed and UVC dwell-time remains under 7 days

BW, birth weight; CLABSI, central line associated bloodstream infection; CVC, central venous catheter; NICU, neonatal intensive care unit; PICC, peripherally inserted central catheter; UVC, umbilical venous catheter; UAC, umbilical arterial catheter; VLBW, very low birth weight.

### 3.2. Studies That Demonstrate an Association between UVC Dwell Time and Risk of CLABSI (Table 2)

Butler O’Hara and coworkers conducted a retrospective large cohort study, including 984 neonates delivered from 1 January 2006 to 31 December 2009 [9]. During the study period, PICC insertion was standardized, and care bundles were introduced. An umbilical venous catheter (UVC) was placed as part of routine care. Neonates were divided into the following groups according to the UVC dwell time: ≤7 days (448 neonates, 45%) and >7 days (536 neonates, 55%). Neonates with a UVC dwell time of ≤7 days, as compared to those with >7 days, had 1.0 and 4.0 CLABSI/1000 catheter days, respectively (p < 0.01). This study also found that CLABSI rates were higher in UVC with a prolonged dwell time as compared to PICC. Authors concluded that CLABSI may be reduced if UVC is replaced with a PICC when a central venous access is still required after 7 days. However, authors acknowledged several limitations: firstly, the mean gestational age, the body weight and the severity of the disease were different between the two study populations. Secondly, neonates exposed to short or prolonged catheter dwell time had also different exposures to central line care practices. This is also confirmed in the more recent literature on CVC, which suggests the relevant impact of line care practices on CLABSI rates.

Zingg and coworkers carried out a very large prospective single-centre cohort study by enrolling 1124 neonates with one or more central lines from 2001 to 2008 in a university hospital in Switzerland [17]. New-borns with PICC, UVC and UAC were included, while those with long-term tunnelled central venous catheters were excluded. A total of 2116 catheters (723 PICCs, 385 umbilical artery catheters and 1008 umbilical venous catheters) were included. The purpose of the study was to determine the occurrence of CLABSI. Median gestational age was 32 weeks and median birth weight was 1943 g and 44% of neonates were VLBW and 18% were ELBW. Median dwell time was significantly different between PICCs and umbilical catheters (7 days for PICC versus 4 days for UVC, *p* < 0.001). The authors also reported that catheter dwell time varied according on birth weight. In fact, neonates weighing ≤ 750 g had a median catheter dwell time twice as long as those weighing more than 2500 g (7 vs. 3 days, *p* < 0.001). A total of 102 CLABSI episodes were reported, with an overall incidence of 8 CLABSI episodes per 1000 catheter days. The median time of a CLABSI episode in neonates with a PICCs and umbilical (arterial or venous) catheters was 7 (IQR, 5–10) and 7 (IQR, 5–8) days, respectively. CLABSI rates were 80/1000 catheter days for PICCs, 3/1000 catheter days for UAC and 19/1000 catheter days for UVCs. The highest CLABSI rates occurred among neonates with a body weight under 750 g. This study also reported a significant reduction in catheter dwell times over the years, identifying risk factors for CLABSIs (i.e., low birth weight and total parenteral nutrition). Authors concluded that dwell time was a relevant risk factor for CLABSIs, especially for umbilical catheters and PICCs that were in the first 7 days from positioning. After 7 days, according to their data, PICCs are less likely to become infected if they have not become infected up to that time. Considering the median PICC dwell time of 7 days, routine PICC replacement was not recommended.

To determine the incidence and potential risk factors for CLABSI in neonates, Yumani carried out a retrospective cohort study by enrolling 196 neonates in a university hospital in Netherlands [18]. Data of neonates with a UVC, UAC, PICC or another CVC (tunnelled and cuffed or non-tunnelled subclavian, jugular or femoral catheter) for at least 12 h and admitted to the NICU from 1 January to 31 December in 2007 were revised. A total of 369 central catheters were included in the study (182 UVC, 103 UAC, 59 PICC, 25 CVC). Median gestational age was 32 weeks. Catheter type and dwell time were considered as possible risk factors for CLABSI. According to the diagnostic criteria of CLABSI used by the CDC before 2008, CLABSI incidence was 18.1 infections/1000 catheter days (95% CI: 13.7–23.8). The median catheter dwell time was 7 days for UVCs, 5 for UACs, 6 for CVCs and 9 for PICCs. The median catheter dwell time until CLABSI occurrence was 8 days for UVCs and UACs, 6 for CVCs and 18.5 for PICCs. Umbilical catheters had higher infection rates than non-umbilical catheters. As a matter of fact, the CLABSI rate per 1000 catheter days was 22.1 for umbilical catheters, 9.5 for CVC and 14.4 for PICC. Moreover, prolonged umbilical catheter dwell time (≥7 days) is associated with an augmented risk of infections, with a rate of CLABSI increasing from 7.4 episodes/1000 catheters days to 27.8. Similarly, non-umbilical catheters were associated the risk of infections when retained for 14 days or more. In conclusion, the authors recommend minimizing catheter dwell time and retaining umbilical catheters for less than one week. However, several limitations of the study should be noticed, including the retrospective design and the relatively small sample size.

Finally, Sanderson and coworkers conducted a retrospective analysis of prospectively collected data [19]. They enrolled 3985 neonates born from 1 January 2007 to 31 December 2009 in 10 Australian NICUs. Neonates were included if they had an UVC or a PICC and were divided into three groups: UVC only (group 1, n = 1392), PICC only (group 2, n = 1317) or both (UVC replaced by PICC, group 3, n = 1276). They reported a total of 403 CLABSI among 6000 catheters inserted. Rates of CLABSI increased from 3.3/1000 UVC days in group 1, to 4.8/1000 PICC days in group 2 and to 16.9/1000 UVC days in group 3. By using life table and Kaplan–Meier hazard analysis, they showed that UVC CLABSI rates increased stepwise to 42/1000 UVC days by day 10, particularly in group 3 (85/1000 UVC days). In contrast to UVC, PICC CLABSI rates remained relatively stable at 12–20/1000 PICC days. When risks were controlled for dwell time, UVCs had a higher adjusted CLABSI risk as compared to PICC. The authors concluded that, in group 3, the elective early removal of UVC before day 4 and its replacement with a PICC would reduce CLABSI risk. The data showed that the risk of UVC-related CLABSI increases progressively within the first week after insertion, and an early UVC removal (within 2 or 3 days of life) is warranted. Furthermore, the risk of PICC-related CLABSI peaked in the second week after insertion and remained unchanged in the third week.

**Table 2:** Studies showing a correlation between UVC dwell time and risk of CLABSI.

Study Design (Ref.)	Study Population	Main Results	Conclusions
Retrospective cohort study *Single centre* [9]	**n = 984** Group 1 (n = 448): UVC ≤ 7 days Group 2 (n = 536): UVC > 7 days	Group 1: **1** CLABSI per 1000 catheter days Group 2: **4** CLABSI per 1000 catheter days *p* < 0.001	CLABSI are reduced when an UVC is replaced with a PICC within 7 days of age
Prospective single-center cohort study *Single centre* [17]	**n = 1124 (2116 CVCs)** Median gestational age: 32 weeks Group 1: 1393 umbilical catheter (median dwell time: 4 days, median time of CLABSI: 7 days) Group 2: 723 PICC (median dwell time: 8 days, median time of CLABSI: 7 days))	Group 1: **3** CLABSI per 1000 catheter days (UACs); **19** CLABSI per 1000 catheter days (UVCs) Group 2: **80** CLABSI per 1000 catheter days Overall incidence 8 CLABSI episode/1000 catheters-day	Dwell time (in both UVC and PICC) correlates with CLABSI. After 7 days, PICCs are less likely than UVC to become infected
Retrospective cohort study *Single centre* [18]	**n = 196** (369 UVC, UAC, PICC or other CVC) Median gestational age: 32 weeks	Group 1 (UVC and/or UAC): 22.1 CLABSI per 1000 catheter days (7.4 if < 7 days, 27.8 if ≥ 7 days) Group 2 (other CVC): 9.5 CLABSI/1000 catheter days Group 3 (PICC): 14.4 CLABSI/1000 catheter days	As compared to PICC, UVC has the highest infection rate. UVC dwell time over 7 days increase CLABSI
Retrospective study (population data prospectively collected) *Multicentre* [19]	**n = 3985** Group 1 (n = 1392): UVC only Group 2 (n = 1317): PICC only Group 3 (n = 1317): UVC + PICC	Group 1: CLABSI **3.3**/1000 catheter days Group 2: CLABSI **4.8**/1000 catheter days Group 3: CLABSI **16.9**/1000 catheter days UVC CLABSI rates increased stepwise to 42/1000 UVC days by day 10	Elective early removal of UVC (prior to day 4) and its replacement with a PICC could reduce CLABSI

BW, birth weight; CLABSI, central line associated bloodstream infection; CVC, central venous catheter; NICU, neonatal intensive care unit; PICC, peripherally inserted central catheter; UVC, umbilical venous catheter; UAC, umbilical arterial catheter; VLBW, very low birth weight.

## 4. Discussion

The issue of risks associated with prolonged central catheter dwell time in newborns is a debated issue in the scientific literature. As mentioned earlier, the need to maintain a safe access to administer drugs and parenteral nutrition for a prolonged time often conflicts with increasing risk of catheter-related infections. Hence, many authors have attempted to assess the maximum dwell time of central catheters to reduce at minimum the risk of infection.

Studies that do not demonstrate a correlation between central catheter dwell time and CLABSI risk have several limitations that affect the applicability of their results. These studies often enroll a limited number of infants [6,15] or they have an imperfect study design [6,14]. It is worth noting that Butler O’Hara’s study that allowed catheters to remain in place for a very prolonged period (up to 28 days). This UVC dwell time is in sharp contrast to the remaining literature, limiting its applicability. Despite an important limitation, we decided to include this study in our review because of its randomized design, very unusual in studies regarding UVCs. For this reason, it was the only study included in the 2017 Cochrane review [13,20].

On the other hand, studies demonstrating the increasing risk of infection after a prolonged catheter dwell time have usually better designs (Section 3.2). They provide more details about population included and the inclusion criteria. Moreover, these studies usually include larger populations and provide information also about care bundle and the way in which proper catheter placement was confirmed [9,17,19]. The largest population was studied by Sanderson and coworker (almost four thousand newborns). Analyzing in detail prospectively collected data, Sanderson demonstrate a positive correlation between CVCs dwell time and CLABSI risk [19].

More recently, a narrative review provides an overview of current knowledge and evidence in the field of UVCs (choice of the device, mode of insertion and care of the UVC, securement, post-procedural X-ray, migration and infective or non-infective complications) [5]. The authors of this review recommend an early planned removal when the clinical indications of UVC placement are no longer present. This way, if a longer central venous access is required, they suggest replacing (within 4 days) the UVC with an epicutaneous-caval catheter (ECC) or with a central ultrasound-guided venous access. The same indication of an early removal is also given by Infusion Nursing Society (INS) guidelines [11]. To prevent infections, they suggest limiting the UVC dwell time to a maximum of 7–10 days but the UVC should be removed earlier (within 4 days) and replaced with a PICC when a central access is still required as an infection prevention strategy.

Although in this review we focused on the CVC dwell time as a risk factor for CLABSI, there are additional ones. In fact, several studies have also recognized prematurity, low birth weight, total parenteral nutrition, comorbidity and male gender as conditions, which increase CLABSI risk. In particular, prematurity seems to be the main risk factor, even more than the dwell time and the birth weight [16,21]. The type of central catheter used also contributes to determine infectious risk. In fact, Levit and coworkers in their study demonstrated that the risk of complications, excluding UVC malposition, were significantly higher with the use of double-lumen UVCs as compared with single-lumen UVCs and that the risk of all UVC-associated complications increased with dwell time, most notably after 16 days of UVC utilization [22].

Several factors contribute to determine the risk of CLABSI (such as gestational age, sex, birth weight, catheter type, catheter dwell time and care bundle) and all should be taken into account.

The latest guidelines recommend limiting the UVC dwell time to a maximum of 7–10 days and the replacement with an ECC even to 4 days if a long need for central access is suspectable. However, this is only a suggestion rather than an imperative indication, as each infant should be critically evaluated according to individual clinical findings and risk factors. Furthermore, not all NICUs may have the same resources (i.e., type of catheter, care bundle). Consequently, clinicians should evaluate the risk–benefit ratio by considering all risk factors, although prematurity and dwell time are the two main risk factors associated with infectious complication.

A scorecard evaluating individual CLABSI-associated risk factors would be helpful to guide decisions regarding CVCs dwell time, as suggested by Vachharajani and his group [23]. The author introduced a questionnaire designed to prompt critical thinking in clinicians regarding the need to replace the UVC with a PICC. In particular, if newborns had specific clinical characteristics (i.e., birth weight > 1000 g or gestational age > 27 weeks, UVC in place on admission to NICU, extubated and tolerating enteral feeds by 72 h of life), care providers were encouraged to leave the UVC for more than 7 days (the recommended ‘safe’ period). On the other hand, the questionnaire also encouraged caregivers to remove the UVC and insert a PICC after day 7 if the neonate was not tolerating 60–70 mL/kg per day of feeds by 5–6 days of age. The score obtained by weighing risk factors could be useful to the clinician in deciding whether in individual cases it is preferable to allow a longer UVC permanence (i.e., for 10 days); alternatively, clinicians would replace UVC earlier with PICC (i.e., UVC for 4 days followed by PICC for 6 days). The score would allow the clinician to make a balanced choice between the two options.

## 5. Limitations

Our review has some limitations. Firstly, the available studies are almost exclusively retrospective in nature and often include very heterogeneous populations; indeed, they often include both full term and preterm neonates with different birth weights.

Furthermore, the number of infants enrolled is often small; this makes it difficult to reveal relatively rare events, such as complications related to catheter dwell time.

## 6. Conclusions

Best-designed studies and updated guidelines suggest limiting UVC dwell time to 7–10 days. However, it would be appropriate to replace the UVC with an ECC within 4 days if a prolonged need for a central line is expected (i.e., in premature neonates who require parenteral nutrition). When possible, single-lumen catheters should be preferred; moreover, appropriate care bundles should be utilized. As previously suggested, a scorecard would be helpful in determining the individual risks of CLABSI and consequently decide the correct individualized catheter dwell time [23].

Although the issue of this review is focused on infectious complications related to CVCs (especially UVCs), we highlight the relevance of non-infectious complications, such as thrombosis and difficulties in removing the CVO, which could be minimized with a closer ultrasound monitoring of the catheter tip.

## Data Availability

The data that support the findings of this study are openly available in PubMed [1,3,4,5,6,7,8,9,11,12,13,14,15,16,17,18,19,20,21,22,23]. Reference [2] is available in PubMed with URL at: https://pubmed.ncbi.nlm.nih.gov/9832755/ (accessed on 25 December 2022). Reference [10] is available with URL at: https://www.cdc.gov/nhsn/pdfs/pscmanual/pcsmanual_current.pdf (accessed on 25 December 2022).
